# Do outcomes of psychiatric hospital treatment differ for migrants and non-migrants?

**DOI:** 10.1007/s00127-021-02103-y

**Published:** 2021-05-24

**Authors:** Kimberley Anderson, Domenico Giacco, Victoria Bird, Michael Bauer, Andrea Pfennig, Antonio Lasalvia, Mirella Ruggeri, Vincent Lorant, Pablo Nicaise, Jacek Moskalewicz, Marta Welbel, Stefan Priebe

**Affiliations:** 1 Unit for Social & Community Psychiatry, WHO Collaborating Centre for Mental Health Services Development, East London NHS Foundation Trust, Newham Centre for Mental Health, Queen Mary University of London, London, E13 8SP UK; 2 Warwick Medical School, University of Warwick, Gibbet Hill Campus, Coventry, CV4 7AL UK; 3 Department of Psychiatry and Psychotherapy, Carl Gustav Carus University Hospital, Klinik und Poliklinik für Psychiatrie und Psychotherapie, Technische Universität Dresden, Universitätsklinikum Carl Gustav Carus, Fetscherstraße 74, 01307 Dresden, Germany; 4 UOC Psichiatria, Azienda Ospedaliera Universitaria Integrata di Verona, Piazzale Aristide Stefani, 1, 37126 Verona, VR Italy; 5 Section of Psychiatry, Department of Neuroscience, Biomedicine and Movement Science, University of Verona, Verona, Italy; 6 Institute of Health and Society, Université Catholique de Louvain, Clos Chapelle-aux-champs, 30 bte 30.15, 1200 Woluwe-Saint-Lambert, Brussels, Belgium; 7 Institute of Psychiatry and Neurology, ul. Sobieskiego 9, 02-957 Warsaw, Poland

**Keywords:** Migration, Europe, Mental illness, Inpatient, Hospital care

## Abstract

**Purpose:**

Providing effective treatment for immigrants is an increasing challenge for mental health services across Europe. Yet, little is known as to whether current practice is associated with different outcomes in migrant and non-migrant patients. We compared outcomes of inpatient psychiatric treatment for migrants and non-migrants in a sample from five European countries.

**Methods:**

Patients with psychotic disorders, affective disorders or anxiety/somatisation disorders admitted to routine psychiatric inpatient treatment were assessed in hospitals in Belgium, Germany, Italy, Poland and the United Kingdom. Treatment outcomes were satisfaction with care during hospitalisation, length of stay, readmission to hospital (any and, specifically, involuntary re-hospitalisation), as well as untoward incidents in a 1-year follow-up period. Outcomes were compared between patients born inside (non-migrants) and outside (migrants) the country of treatment, through mixed regression models.

**Results:**

Across all sites, 985 migrant patients and 6298 non-migrant patients were included. After accounting for the influence of confounding patient characteristics, migrants reported significantly lower treatment satisfaction, but there were no significant differences for length of stay and re-hospitalisations, in general and involuntary ones. Migrants had a lower rate of suicide attempts, but there was no significant difference in other types of untoward incidents in the year following the index admission.

**Conclusion:**

The study suggests that migrants are less satisfied with their hospital treatment, there is no evidence that routine inpatient care as currently provided results overall in poorer objective outcomes for migrants than in non-migrant populations.

## Introduction

Based on Eurostat data for 2018 at a time when the European Union comprised 28 countries, out of its 512 million people, 62.5 million (12.2%) were migrants defined as people born outside the country of residence [1]. Subsequently, the provision of healthcare services to such populations has been a source of continuous debate over recent years [2–5]. Health policies for migrant populations recognise their less favourable outcomes in terms of employment, education and social inclusion, indicators which are frequently associated with health outcomes later on [6]. Migrant populations can have different expectations of care, different preferences for treatments and different appraisals of received interventions than non-migrants. This may be as a result of a lack of trust in services, preconceived notions about healthcare staff in the host country, distinct explanatory models of disorders, the experience of specific staff attitudes or actual differences in the provided treatments [5, 7]. For some migrants, there is also the possibility of having experienced traumatic life events, post-migration stress and acculturation and ethnic discrimination [8, 9], all of which can cumulatively influence their perception of care [2, 3].

Disparities in outcomes between migrants and non-migrants may exist particularly in mental health care [9, 10]. Findings from studies that compare mental health outcomes are inconsistent, and results amongst migrants include an increased risk of involuntary admission [10], lower rates of suicide attempts [11], and lower uptake of psychological services and medication use [2]. Yet, there is a lack of large-scale research on whether outcomes of psychiatric treatments are truly different for migrant groups, and this applies to objective outcomes as well as patients’ subjective appraisal of it [12]. Patient satisfaction is known to be a key indicator of quality of care [13, 14], and is described as “an expression of the gap between the expected and the perceived quality of a service [11, page 69]. Higher levels of satisfaction with inpatient care have been shown to be associated with lower readmission rates to hospital and lower disability levels within 1 year [15–17], better adherence to medication and treatment, and a reduction in psychotic symptoms up to three 3 years later [16, 18, 19]. For migrants specifically, satisfaction with mental health care in their country of residence remains poorly understood.

Given the lack of large-scale findings about outcomes of routine inpatient care amongst migrants as compared to non-migrants, this study compares outcomes of psychiatric inpatient care in a sample from five European countries, using different outcome criteria: patient satisfaction with treatment in the hospital as a subjective outcome; and as objective indicators length of stay in hospital, and re-hospitalisations in general and involuntary re-hospitalisations as well as untoward incidents in the year following the index admission.

## Methods

### Study design

Data from the present study are part of an observational, longitudinal study conducted across five European countries (Belgium, Germany, Italy, Poland and the UK). Within each country, hospitals were purposively selected to include a range of rural, urban or semi-urban locations and included 57 in total (for more details and other aspects of this study, see [14, 18–20]).

### Participants

Participants were included in the study if they met the following inclusion criteria: (1) aged 18 years or older; (2) International Classification of Disease-10 (ICD-10) diagnosis of psychotic disorder (F20–29), affective disorder (F30–39) or anxiety/somatisation disorder (F40–49), (3) hospitalised in a general adult psychiatric inpatient unit; (4) sufficient command of the language of the country to provide written informed consent and understand the research questions and (5) capacity to provide informed consent. Individuals with a diagnosis of organic brain disorders and cognitive impairment too severe to enable completion of the study assessments were excluded. As pre-specified in the analysis plan for the study, we considered as migrants all patients who reported their country of birth as different from the country in which they were recruited.

### Procedure

Patients consecutively admitted to adult psychiatric wards between October 2014 and December 2015 were screened to determine eligibility for the study, and all eligible patients were approached by a trained researcher within 48 h of admission. The researcher provided information about the study and obtained written informed consent prior to completing the questionnaire containing the study measures. Where an individual lacked capacity to provide informed consent, they were re-approached at a later stage in their admission. The questionnaire was completed in a face-to-face interview with the researcher, and included data on socio-demographic characteristics, clinical outcomes and satisfaction with inpatient care. Age, gender, country of recruitment, partnership status, education and employment were assessed as socio-demographic characteristics; and primary psychiatric diagnosis at discharge on ICD-10, whether it was a first time admission, whether it was a voluntary admission and severity of illness (as measured by the Clinical Global Impression severity scale, CGI; [21]) as clinical characteristics. Information relating to psychiatric diagnoses at discharge, severity of illness and details of the admission including length of stay and formal status, were obtained from medical records (where available) or from the treating clinician. To avoid the possibility for social desirability in treatment satisfaction ratings, researchers collecting data were external to the patients’ clinical team, and satisfaction scores were kept confidential. Follow-up data for 1 year following the index admission were obtained from medical records in England and Italy, with phone or personal interviews being conducted to complement the data. In remaining countries, all outcome data after 1 year were collected by interviewing patients either by phone or face-to-face.

### Outcome measures

We analysed all outcomes assessed in the study for the total sample. Measures were:

1. satisfaction with inpatient care, as measured using the Client Assessment of Treatment Scale (CAT; [14, 22, 23]). The CAT is a seven-item measure, in which participants rate their satisfaction with different elements of their inpatient treatment. Scale items include: “Do you believe you are receiving the right treatment/care for you?”, “Does your therapist/case manager/key-worker understand you and is he/she engaged in your treatment?”, “Are relations with other staff members pleasant for you?”, “Do you believe you are receiving the right medication for you?”, “Do you believe the other elements of treatment/care here are right for you?”, “Do you feel respected and regarded well?”, and “Has treatment/care here been helpful for you?”. For each item, the person rates their response on an 11-point Likert scale ranging from 0 (not at all) to 10 (entirely). The CAT has been widely used to assess patient satisfaction with inpatient treatment, and has been validated for use with psychiatric inpatients and across different countries [14];
2. the length of stay of the index admission in days;
3. any readmission to hospital in the following year and specifically any involuntary readmission (each binary: yes/no), and
4. experience of untoward events including death, suicide, suicide attempt, serious side effects, accused of a crime, victim of physical violence (each binary: yes/no).

All these measures had been pre-specified in the analysis plan.

### Data analysis

Baseline characteristics were compared using mixed effect models, adjusted for the effect of clustering amongst hospitals. For the CAT, where individuals had missing data for two items or fewer, the mean of the remaining items was used to replace the missing items. Where more than two items were missing, the person was excluded from the analysis. Rehospitalisation accounted for anyone who had a negative treatment outcome, including participants who were rehospitalised once or multiple times, who were hospitalised for the full year or who died during the follow-up period.

Mixed effects models were employed to analyse the outcomes: linear regression for continuous outcomes (mean satisfaction score and length of stay) and logistic regression for binary outcomes (re-hospitalisation and untoward incidents). Analyses accounted for the differences in socio-demographic and clinical characteristics between the two groups of patients, which were included in the models. A random effect was incorporated to account for the clustering of patients within hospitals. Odds ratios (OR), confidence intervals (CIs) and *p* values for each outcome of migrants and non-migrants were reported. The level of statistical significance was set at *p* < 0.05 for all the analyses, and were conducted using Stata v14.

## Results

### Sample characteristics

Of the 24,645 patients screened, 10,291 were excluded as ineligible: 520 of them because of insufficient command of the language of the host country. A total of 14,354 patients were deemed eligible and approached to take part in the study, of whom 7777 completed the baseline assessments. At this stage, a further 475 patients either withdrew (*n* = 75) or were excluded because of an ineligible diagnosis (*n* = 372) or other reasons (*n* = 28), which resulted in a baseline sample of 7302 patients (for details, see [18]). For 19 patients, data on the migrant status were missing, so that they were excluded from any further analysis. A total of 6298 (86.3%) patients reported being born in the same country, 985 (13.5%) were classified as migrants: Belgium 123 (17% of the total sample in Belgium), Germany 149 (21%), Italy 136 (12%), Poland 21 (2%) and UK 556 (21%). Socio-demographic and clinical characteristics of the total sample and each group are shown in Tables 1 and 2.

**Table 1:** Socio-demographic characteristics of the total sample and of migrant and non-migrant patients

	Total sample (*n* = 7302)		Migrant (*n* = 985)		Non-migrant (*n* = 6298)	
	*n*	%	*n*	%	*n*	%
Age (> 40 yes)**	42.4	14.3	39.5	12.8	42.9	14.4
Gender (female)*	3475	47.6	446	45.3	3017	47.9
Marital status*						
Married	1529	20.9	220	22.3	1306	20.7
Single/unmarried	3922	53.7	516	52.4	3397	53.9
Widow	258	3.5	22	2.2	235	3.7
Divorced	869	11.9	128	13.0	741	11.8
Co-habiting	309	4.2	24	2.4	284	4.5
Separated	352	352	66	6.7	285	4.5
Education**						
Primary education	1259	17.2	122	12.4	1134	18.0
Secondary education	2983	40.9	351	35.6	2626	41.7
Higher education	2907	39.8	484	49.1	2417	38.4
Other	71	1.0	9	0.9	62	1.0
Unknown	45	0.6	14	1.4	29	0.5
Employment						
Unemployed	3201	43.8	495	50.3	2703	42.9
Paid employment	1993	27.3	247	25.0	1739	27.6
Voluntary employment	95	1.3	12	1.2	83	1.3
Sheltered employment	75	1.0	6	0.6	68	1.1
Student	384	5.3	65	6.6	316	5.0
Housewife/husband	261	3.6	42	4.3	218	3.5
Retired	928	12.7	63	6.4	863	13.7
Other	338	4.3	52	5.3	286	4.5

All variables adjusted for hospital

*p ≤ 0.05 **p ≤ 0.001

**Table 2:** Clinical characteristics of the total sample and of migrant and non-migrant patients

	Total sample (*n* = 7302)			Migrant (*n* = 985)			Non-migrant (*n* = 6298)		
	*n*		%	*n*		%	*n*		%
Diagnosis at discharge**									
Psychotic disorders	2798		38.3	434		44.1	2356		37.4
Mood disorders	3057		41.9	402		40.8	2648		42.0
Anxiety/somatisation disorders	973		13.3	97		9.8	872		13.8
Other	448		6.1	52		5.3	396		6.3
First admission (yes)**	2435		33.3	374		38.0	2058		32.7
Involuntary (yes)**	1635		22.4	321		32.6	1281		20.3
Severity of illness (mean, SD)	4.31		1.18	4.45		1.2	4.28		1.18

All variables adjusted for hospital

*p ≤ 0.05 **p ≤ 0.001

Migrant and non-migrant patients showed differences in socio-demographic and clinical characteristics and most of these differences were statistically significant, i.e. age, gender, marital status, education, diagnosis at discharge, and whether it was a first or involuntary admission. The migrants were younger, had more often a higher education, and were more often unemployed than non-migrants. Moreover, psychotic disorders were more frequent amongst migrants, and migrants had more often been hospitalised for the first time and also more often involuntarily.

### Outcomes

Outcome data for the total sample and for migrant and non-migrant patients are shown in Table 3.

**Table 3:** Unadjusted and adjusted outcomes of mixed regression model

	Total sample (*n* = 7302)		Migrant (*n* = 985)		Non-migrant (*n* = 6298)		Adjusted*		
	Mean	SD	Mean	SD	Mean	SD	OR	CI 95%	*p*
Satisfaction with treatment (CAT)	7.28	2.15	7.14	2.38	7.31	2.12	0.188	0.032–0.344	0.018
Length of stay (days)	39.4	49.7	43.0	55.7	38.8	48.6	1.04	0.732–1.015	0.289

	*n*	%	*n*	%	*n*	%	OR	CI 95%	*p*
Any re-hospitalisation (within 1 year following index admission)	2344	32.1	288	29.2	2050	32.6	0.89	0.757–1.058	0.193
Involuntary re-hospitalisation (within 1 year following index admission)	412	17.6	80	27.8	329	16.1	0.79	0.539–1.184	0.264
Untoward event	1125	15.4	166	16.9	959	15.2	0.85	0.682–1.058	0.144
Death	77	1.1	6	0.6	71	1.1	0.647	0.272–1.539	0.325
Suicide	46	0.6	4	0.4	42	0.7	0.715	0.243–2.104	0.542
Attempted suicide	445	6.1	49	5.0	396	6.3	0.687	0.478–0.988	0.043
Serious side effects	194	2.7	17	1.7	177	2.8	0.84	0.475–1.489	0.554
Accused of crime	411	5.6	69	7.0	341	5.4	0.86	0.626–1.190	0.370
Victim of violence	413	5.7	71	7.2	341	5.4	1.06	0.774–1.441	0.727

All variables adjusted for hospital

*Adjusted for significant baseline characteristics: age, marital status, education, diagnosis at discharge, whether it was a first admission and whether the index admission was voluntary

Using mixed regression models, adjusting for the hospital that patients were recruited at and for socio-demographic and clinical characteristics, satisfaction with care showed a statistically significant difference between the two groups. Migrants reported a significantly lower satisfaction with treatment than non-migrants, albeit small. There was no significant difference in re-hospitalisations, neither in general nor specifically in involuntary ones, within 1 year following the index admission. The unadjusted rates suggest that migrants had many more involuntary re-hospitalisations, but the difference is not statistically significant when all the differences in baseline characteristics between the two groups are considered in the analysis. During the same 1-year follow-up period, migrants had a lower rate of suicide attempts. The difference in unadjusted rates of suicide attempts between the two groups was small, but—again, after adjusting for baseline characteristics—statistically significant. There was no significant difference in the rate of any of the other assessed untoward incidents.

## Discussion

### Main findings

This large multinational study assessed outcomes of routine psychiatric inpatient treatment in migrants and non-migrants. After adjusting for confounding patient characteristics, a small, but statistically significant difference was found between migrants and non-migrants with regard to their satisfaction with treatment, but not regarding most of the objective outcome indicators, i.e. length of stay, any re-hospitalisation or specifically an involuntary re-hospitalisation during the year following the index admission. With respect to untoward events during the same period, there was no statistically significant difference either, other than in suicide attempts which were slightly less frequent amongst migrants.

### Strengths and limitations

In this study, the sample of more than 7,000 patients across 5 European countries was large and provided considerable statistical power to adjust differences between migrants and non-migrants for potentially confounding characteristics of the patients. This is essential for such a comparison since migrants and non-migrants differ in a range of socio-demographic and clinical characteristics, and these characteristics need to be taken into account in a meaningful statistical analysis [5, 7, 13, 24]. Patients were studied in routine care and outcomes were not influenced by the research process. The findings can, therefore, be assumed to truly reflect what happens in routinely provided care, at least in the 57 participating hospitals. A further strength is that both subjective and objective outcome criteria were assessed, providing a more comprehensive picture than a focus on only one or two outcomes would have done. Satisfaction with care in this study was assessed within a few days of admission to hospital, so that the study was able to capture those patients only admitted for a short time. Such timely reporting of satisfaction also avoids a retrospective appraisal of care [15] that typically differs when asked at or following discharge. The follow-up rate was high in both groups so that the 1-year outcomes in migrants and non-migrants are unlikely to have been influenced by substantial selection biases.

The study nonetheless has several limitations. First, patients who could not speak the language of the host country sufficiently to complete assessments were excluded, which may have particularly affected recently arrived migrants. Though this number was only a relatively small amount compared to eligible patients who were approached, nonetheless, given the analysis focussed on the comparison of experiences between migrants and non-migrants, a significant proportion of those who might otherwise be eligible were not included. Language and culturally bound difficulties in the treatment process could lead to lower satisfaction in migrants compared to non-migrants. Second, we did not distinguish between migrant groups based on their country of origin, host country, legal status and length of stay in the host country, and all analyses, therefore included a large and heterogeneous sample of migrants. Specifying groups by country of origin and host country alone would have resulted in a large number of relatively small sub-samples. Such smaller sub-samples would have provided little statistical power to detect differences and made it impossible to adjust the analysis of differences for confounding socio-demographic and clinical characteristics, which, however, is necessary to arrive at sound conclusions. Third, although we included a substantial total number of hospitals and conducted the study in five countries, contextual factors may have influenced the findings and the results cannot necessarily be generalised to all other hospitals in the same or different countries in Europe. Fourth, we did not assess details of the complex and variable treatments themselves, and cannot conclude as to whether specific treatment components are associated with different outcomes. Finally, although we assessed several outcome criteria, there are further relevant criteria that were not considered such as specific symptoms and clinical improvement during treatment, although the global clinical severity as assessed by the clinician was considered and adjusted for in the analysis of differences. Length of stay may be seen as a proxy for the speed of clinical improvement since patients are usually discharged only when they are seen as having sufficiently improved. Yet, a direct measure of clinical improvement might still have yielded a different result.

### Implications

This study provides a reliable answer as to whether migrants and non-migrants in Europe—considered overall as one group—differ in their satisfaction with inpatient treatment and in some relevant objective outcomes up until a year after the index admission. Clinicians and other stakeholders may have the impression that in the daily practice of a given hospital migrants have poorer outcomes, possibly because they express a lower satisfaction with treatment when receiving it. Yet, this study has found no evidence for generalised statements about differences in objective outcomes. Even a large difference in involuntary re-hospitalisations was not statistically significant, once confounding patient characteristics were considered, so that it seems to be explained by characteristics other than the migration status. The only exception was rates of suicide attempts which were less frequent amongst migrants. The relatively small difference is consistent with previous findings about lower suicide rates amongst migrants [11].

A clear understanding that there is no evidence to suggest that migrants are staying longer in psychiatric inpatient facilities, or are experiencing a greater number of untoward events or readmissions to hospital, should be considered in debates around current practice and good practice for mental health care in Europe [22]. The lack of statistically significant poorer objective outcomes of migrant patients is particularly noteworthy since migrants do have lower satisfaction levels with hospital treatment, and a poorer satisfaction with inpatient treatment tends to be linked with poorer long-term outcomes [16]. Yet, migrants seem to have similar long-term outcomes despite not being as satisfied with their inpatient treatment as non-migrants are.

Whilst the findings on objective outcomes appear reassuring, migrants reported lower satisfaction with hospital treatment itself. The difference between migrants and non-migrants was statistically significant and held true when adjusted for potential confounders. Nevertheless, its size was small, and on its own might not be seen as a reason to consider major changes in practice. At the same time, all practice should aim to improve patient satisfaction for all groups anyway. Various suggestions for how to improve practice in the treatment of migrants in psychiatric services have been published and might be considered in attempts to improve the quality and appropriateness of care [5, 7, 13]. If satisfaction with inpatient treatment can be improved for significant numbers of migrant patients, that will be a considerable achievement in itself. It then needs to be tested whether such improved satisfaction will also be associated with more favourable objective long-term outcomes.

For the evaluation of routine care, one may conclude that similar outcome assessments should be conducted routinely to provide feedback on outcomes and to guide policies, but that the numbers should be large enough to allow for the adjustment of analyses for confounding patient characteristics. Research may focus on treatment processes and test specific interventions for defined groups to improve their treatment satisfaction, and explore whether this may lead to even more favourable objective outcomes.

## Conclusion

The outcomes of psychiatric healthcare provided for migrants and non-migrants across Europe still have a limited and inconsistent evidence base. This study has added to that evidence base and suggests that—overall—migrant patients in Europe are less satisfied with their psychiatric inpatient treatment than non-migrants, but do not appear to have poorer objective outcomes. Whilst specific groups of migrants in certain contexts (nuances we recommend be investigated in future research) may well have different outcomes of psychiatric inpatient treatment than non-migrants, in general, statements that psychiatric hospital care in Europe may fail migrant patients do not appear justified based on the data obtained in this study.

## Data Availability

The authors are willing to share anonymised data upon reasonable request. Access to data needs to be subject to a formal data sharing agreement to ensure that any other researcher accessing the data follows appropriate standards in terms of information governance as stipulated with the national Ethics Committees. Interested researchers can contact v.j.bird@qmul.ac.uk to initiate the process.
